# Outcome of early active mobilization after extensor tendon repair

**DOI:** 10.4103/0019-5413.41859

**Published:** 2008

**Authors:** Narender Saini, Mohan Sharma, VD Sharma, Purnima Patni

**Affiliations:** Department of Orthopedics, SMS Medical College and attached Hospitals. Jaipur, India; 1 Kiran Nursing Home, Mandawar Road, Mahua (Rajasthan), Jaipur; 2 SMS Hospitals, Jaipur; 3 Head of Hand Surgery Unit, SMS Hospital, Jaipur

**Keywords:** Extensor tendon injuries, static splint, early active mobilization, rehabilitation after extensor tendon repair

## Abstract

**Background:**

Traditionally the repaired extensor tendons have been treated postoperatively in static splints for several weeks, leading to formation of adhesions and prolonged rehabilitation. Early mobilization using dynamic splints is common, but associated with many shortcomings. We attempted to study the results of early active mobilization, using a simple static splint, and easy-to-follow rehabilitation plan.

**Materials and Methods:**

In a prospective study 26 cases of cut extensor tendons in Zone V to VIII were treated with primary or delayed primary repair. Following this, early active mobilization was undertaken, using an easy-to-follow rehabilitation plan. The results were assessed according to the criteria of Dargan at six weeks and one year.

**Results:**

All the 26 patients were followed up for one year. 20 out of 26 patients were below 30 years of age, involving the dominant hand more commonly (16 patients, 62%). Agriculture instruments were the most common mode of injury (13 patients, 50%). The common site for injury was extensor zone VI (42%, *n* = 11).

**Conclusion:**

Rehabilitation done for repaired extensor tendon injuries by active mobilization plan using a simple static splint has shown good results.

## INTRODUCTION

Extensor tendon injuries are very common (61.3%)^1^ and are reported to be more common than flexor tendon injuries.^2^ The extensor tendons are predisposed to laceration because of their superficial location on the dorsum of the hand and minimal amount of subcutaneous tissue between the tendons and the overlying skin. This anatomic feature also predisposes the extensor mechanism to more complex tendon injuries, including abrasion, crush, and avulsion of extensor tendons. These injuries are often associated with skin loss.

Rehabilitation after repair of extensor tendon injuries has been less addressed in the literature than that of flexor tendons.^3^ Repaired extensor tendons are immobilized postoperatively in static splints for several weeks. When the splints are removed, extensor lag may occur at the metacarpophalangeal (MP) or interphalangeal (IP) joints, and composite IP and MP flexion is often impossible because of tendon adhesions.^4^,^5^ Early mobilization programs for these injuries have been used for many years by several surgeons and therapists.^3^,^5^–^11^ They used dynamic splinting, similar to that commonly used for flexor tendon injury rehabilitation, particularly following repair in Zone V-VIII (Kleinert and Verdan).^12^ The use of dynamic splint was cumbersome and limited to centers having adequate facilities to manufacture the splint. Thus a need was felt for a simple splint and an easy-to-follow rehabilitation program without the aid of a therapist, so that good functional results could be obtained in areas where minimal facilities were present.

We evaluated the results of a simple static splint used after extensor tendon repair in Zone V-VIII (Kleinert and Verdan),^12^ with early active mobilization rehabilitation plan.

## MATERIALS AND METHODS

This prospective study included 26 cases of fresh extensor tendon injuries that were treated from March 2004 to December 2004 at our institution. Patients were evaluated for final results at six weeks, but were followed for a period of one year. We included both simple and complex injuries. A complex injury is one that has associated injuries, like a fracture and open joint.

### Operative procedure

Where conditions permitted, primary repair of extensor tendons (within 6-12 h) in Zone V to VIII was done (*n* = 14), whereas delayed primary repair was done in the rest. Depending on the circumstances and age of the patient brachial plexus block or general anesthesia was used. Associated bony injuries were fixed with K-wires in 12 cases, whereas in two cases, metacarpophalangeal (MP) joint capsulorraphy was done. Explored cut tendons were minimally freshened and MCP end-to-end repair of the cut tendons was done using Polypropylene 4-0, in children and 3-0 in adults. The suture technique applied varied according to the level of repair and thickness of tendons at that level.

**Figure 1:**
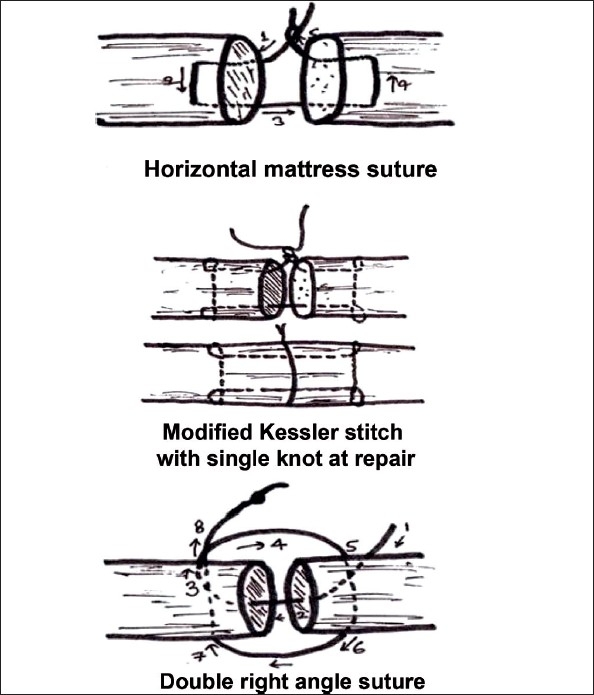
Line diagram showing Horizontal mattress, Modified Kessler, and Double right angle sutures.

In Zone V Horizontal mattress (four cases), in Zone VI Modified Kessler (11 cases) and in Zone VII-VIII Double right angle (11 cases) suture techniques were used. [Figure 1]

Skin closure was done with Polyethylene 3-0. A Plaster of Paris slab was applied volar ward with wrist dorsiflexed to 45° and the slab extended distal to the MP joints, which were kept in full extension the proximal interphalangeal (PIP) and distal interphalangeal (DIP) joints were left free.

### Rehabilitation protocol

**Figure 2:**
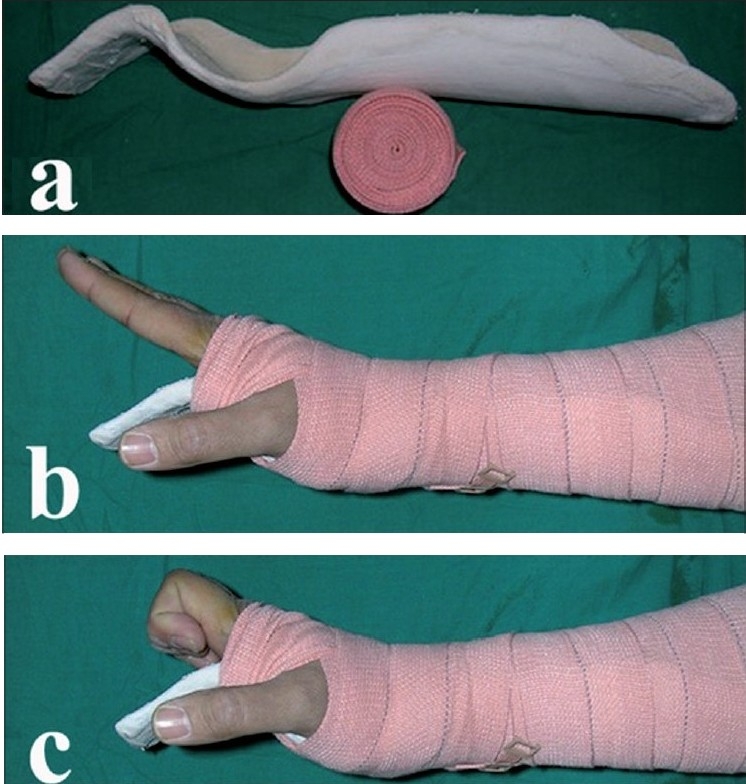
(a) The splint. (b) Combined IP and MCP joint extension exercise. (c) IP joint flexion with MCP joint extension exercise

On the first postoperative day the slab was removed and splint was applied. The splint prepared with Plaster of Paris bandage, was based on Norwich regimen.^13^ The wet plaster bandage was molded over the volar side of the limb with wrist in 45° of dorsiflexion and MP joints flexed at least 50° [Figure 2a]. For thumb injuries the carpometacarpal (CMC) and MP joint were kept in neutral position. The splint was secured with the help of crepe bandage. Controlled active mobilization was begun on the first postoperative day. The patient was instructed to carry out two exercises actively (1) Combined IP and MP joints extension [Figure 2b], and joint extension with IP joint flexion [Figure 2c].

In case of thumb injuries IP joint actively flexed to about 60°. The exercise frequency of the above two exercises was limited to each exercise four times in one session and four sessions each day for the first four weeks postoperatively. For easy comprehension of the patient he was taught the formula of 4×4×4.

On fifth to sixth postoperative day patients were reviewed for dressing. If dressing was clean, the patients were again reviewed on the 10^th^ to 12^th^ day for suture removal. In cases with infection offending sutures were removed and dressing done and the patient was reviewed every two to three days for dressing till the infection cleared. Patients were instructed to continue active mobilization as advised even in the presence of infection.

At four weeks postoperatively X-rays were done in those cases with bone injury and if clinical signs of union of the fracture were present the K-wires were removed. The patients were evaluated for extension lag. If extensor lag was greater than 30° the splint was continued to be worn for another two weeks and original exercises were continued but with unlimited frequency. If no extensor lag was found or the lag was less than 30° the splint was discarded during the day and worn at night for a further two weeks.

After four weeks previous exercises were replaced with gentle flexion of MP joints and IP joints steadily increasing to full flexion and power grip. This fist-making was done with unlimited frequency during the day.

At six weeks the splint was completely removed and extensor strengthening exercises were advised, like flexion of IP joints and active extension and flexion at MP joints with wrist neutral to improve the excursion of repaired extensor tendon. This was done with unlimited frequency. Patients were advised for facilitated gestures requiring full extension of the digits e.g. gestures of namaste and salute.

If the scar was found adherent it was mobilized with lanolin massage three times a day. The patients were encouraged to do their activities of daily living with the injured hand.

The final assessment of progress was done with Dargan Criteria as given in Table 1 below.^14^

**Table 1:** Dargan criteria^14^

Dargan criteria	
EXCELLENT	-NO EXTENSION LAG
	-NO FLEXION LAG
GOOD	-EXTENSION LAG < 15°
	-NO FLEXION LAG.
FAIR	-EXTENSION LAG 16° TO 15°.
	-PULP TO PALM DISTANCE < 2CM.
POOR	-EXTENSION LAG > 45°.
	-PULP TO PALM DISTANCE > 2CM.

## RESULTS

**Figure 3:**
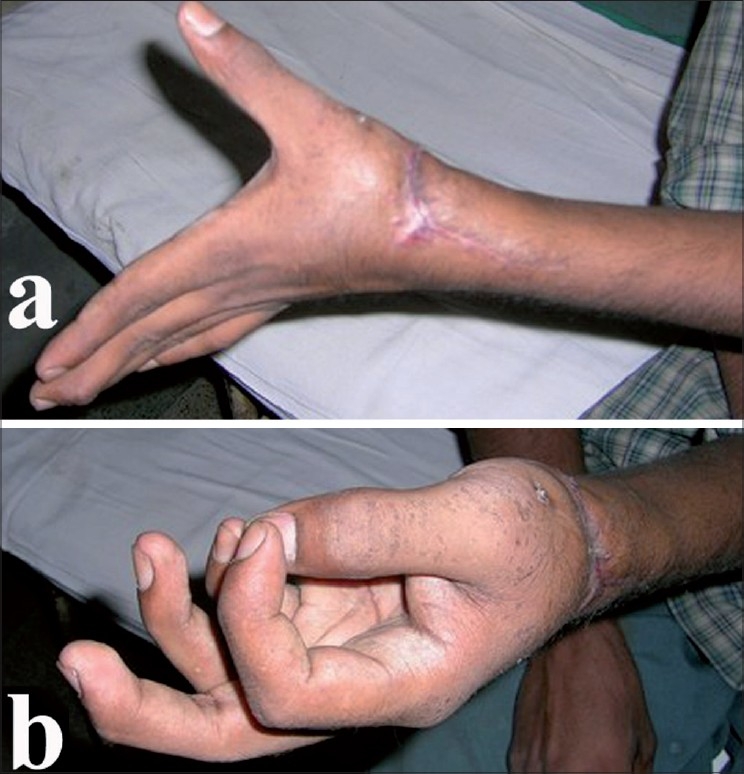
24-year-old male with sword cut ECRL, ECRB and EPL in Zone VII showing excellent results with early active mobilization

**Figure 4:**
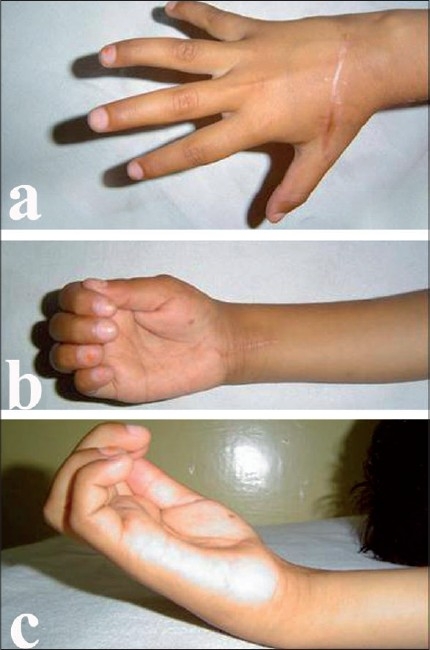
Five-year-old female with fodder-cutting machine injury with cut APL, EPB, ECRL, ECRB, EPL, EDC and EI in Zone VI with also cut FDS/FDP to all fingers showing excellent results with early active mobilization

Twenty-six patients were followed up for a period of one year. There were 19 males and seven females with age ranging between 3 to 55 years (Mean 20.8 years). The dominant hand was involved in 62% (*n* = 16) cases. The majority of the patients were either school-going children, or sedentary workers. The most common mode of injury 50% (*n* = 13) was hand entrapped in fodder-cutting machine. The nature of injury was sharp cut in 81% (*n* = 21) patients, with crush injury seen in only 19% (*n* = 5) cases. The most common site of injury was extensor Zone VI 42% (*n* = 11). The most commonly involved tendons were extensor digitorum communis (EDC), extensor indices (EI) and extensor pollicis longus (EPL). Fifteen per cent (*n* = 4) patients had single tendon involvement, and all of them showed excellent results, whereas 85%, (*n* = 22) had multiple tendon involvement and 91% of them showed excellent results. Of all cases, 14 cases were operated within12 h of injury. This series showed that delay in treatment is not the cause of poor results. Moreover, 100% excellent results were seen in cases developing mild and severe infection whereas non-infected cases resulted in 91% excellent results [Figure 3]. This was because of the fact that infected cases continued with the mobilization protocol despite infection and still gave good results. But amongst the non-infected cases, patients with associated injuries gave poor results. The most common complication was adherent scar in 31% (*n* = 8) patients, and joint stiffness in 8% (*n* = 2) patients. In overall assessment at the end of six weeks, 73% (*n* = 19) patients showed excellent result [Figure 4], 19% (*n* = 5) patients showed good results and 8% (*n* = 2) patients showed poor results. At the end of one year of follow-up five patients with extensor lag improved and thus 92% (*n* = 24) showed excellent results and 8% (*n* = 2) showed poor results. Tendon rupture was not noted in any patient, and no tenolysis was required. No re-repair was needed.

## DISCUSSION

Extensor tendon injuries despite being more common, have received relatively less attention as compared to flexor tendon injuries. Injuries to the extensor mechanism, by contrast may seem relatively simple to treat, but this is not so. The management of these injuries demand the same skill and knowledge as required for flexor tendon injuries.^15^,^16^ On the dorsum of the hand and fingers there is a relative lack of soft tissue, therefore adhesions of the tendon to skin are common, the bone and joints being very close to the dorsal surface are injured concurrently with extensor tendons. These tendons are extra-synovial in most parts except under the extensor retinaculum, they have no vinicula, their blood supply is segmental, arising from the surrounding soft tissue and paratenon. Extensive dissection devitalizes these tendons and promotes scarring and adherence to adjacent structures.

In our series 77% (*n* = 20) of the patients were < 30 years of age. These findings are not consistent with other series where young adults between 18-30 years were more affected.^5^,^15^ This inconsistency is because of the fact that 50% of the injuries were caused by fodder-cutting machines in young school-going children, whereas in other series industrial accidents and road traffic accidents were the most common cause. Males were more often affected than females with M:F = 19:7. These findings are consistent with a ratio of 13:2 in Carla *et al.*^3^ and 24:1 in Pandey and Goyal.^17^ The causative agent has varied in various series depending upon the predominant occupation in the area and location of the hospital. Stuart, 1956, reported a study of 130 patients in whom the injury to the extensor tendon was located over the metacarpal heads; his patients were workers in gold mines and injury was due to sharp edges of quarts crystals. India being an agriculturally predominant country, it's quite understandable that in the present series 50% (*n* = 13) of the injuries resulted form agriculture instruments. The fodder-cutting machine was the main culprit. Mainly children with a rural background were involved (38%) (*n* = 9).

In the present series 30% (*n* = 8) of the injuries were on the radial aspect of the hand and forearm, as these regions were more prone to injury while working. The dominant hand was more commonly involved. In the series of Slater RR, Sacramento^5^ the dominant hand was involved in 18 out of 22 cases (82%).

Twelve cases in our series were complex injuries (46%); despite this fact, the early mobilization protocol showed excellent results in 92% (*n* = 24) cases. This is in contrast to the series of Sylaidis and Logan,^13^ where most of the cases were of simple tendon injuries.

Extensor tendons have been divided into different zones by various authors. Kleinert and Verdan^12^ had divided the whole of the dorsum of the hand, wrist and lower forearm in eight zones. Bunnell had divided it into six zones. In the present series we used the classification of Kleinert and Verdan. Forty-two per cent (*n* = 11) injuries were in Zone VI and 35% (*n* = 9) in Zone VII. This observation is understandable since these two zones are vulnerable to trauma.

Amongst the tendons affected, EDC (81%) (*n* = 21) was most commonly affected in our series, EI (46%) (*n* = 12), and EPL (31%) (*n* = 8) were the next commonest. These findings are consistent with the findings of Slater and Bynum^5^ where EDC was affected in 27 cases, EPL, EI and extensor pollicis brevis (EPB) in six each, abductor pollicis longus (APL) in five, extensor digitorum minimus (EDM), extensor carpi radialis brevis (ECRB) in four and extensor carpi radialis longus (ECRL) in three cases.

Flynn^18^ advocated repair of tendons in clean sharp-cut wounds up to 24 h after trauma, but only within the first 6 h if the wound was crushed or contaminated. In the present series 14 patients (54%) reported within the first 12 h, 11(42%) within 24 h and one (4%) after two days of injury. In 14 cases primary repair was done and in the remaining patients delayed primary repair was done. In 21 patients the wound was clean and tendons were sharp cut, whereas in five cases crushing was present. In all these cases there was normal wound healing except one patient who had a severe infection.

In the present series three (11.5%) cases developed superficial infection, this improved after antibiotics and regular dressings. This did not affect the final outcome. One patient developed severe infection but fortunately after alternate day dressings and antibiotics the final result in this patient was excellent.

The battle against adhesions is an old one and surgeons have now felt that early motion of repaired tendons should have a preventive effect on the formation of the limiting adhesions. Viering^19^ was the first to demonstrate that tendon healing was influenced by motion of the tendon. He reported that fibers of the repaired tendon lined up in a row parallel to the line of pull. Gilberaman^20^ said that early motion increases total DNA content during repair and causes reorientation of peritendinous vessels to more natural longitudinally oriented pattern. Taking lead from many such findings, various attempts were made at early mobilization of the tendons using various types of splints. Unfortunately, while many reports using dynamic splints were encouraging, the technique was both expensive and cumbersome and required frequent input from an experienced capable hand therapist. In our series we tried to overcome these disadvantages, by using a static splint, and an easy-to-follow rehabilitation plan, without the use of a therapist, and still giving equally comparable results.

In the repair of extensor injuries over the wrist, the extensor retinaculum has been an issue of debate. While few authors like Bunnell,^21^ advised excising it completely, others like Lister^23^ advocate that it should be preserved to prevent ugly bowstringing on the wrist and painful dislocation on pronation and supination. Taking a middle path Blue, Spira and Hardy^22^ believed that a portion of overlying extensor retinaculum should be unroofed but every effort should be made to retain a pulley. In the present series retinaculum over the repair was partially unroofed. Full range of movement was obtained without any bowstringing and adhesions.

**Figure 5:**
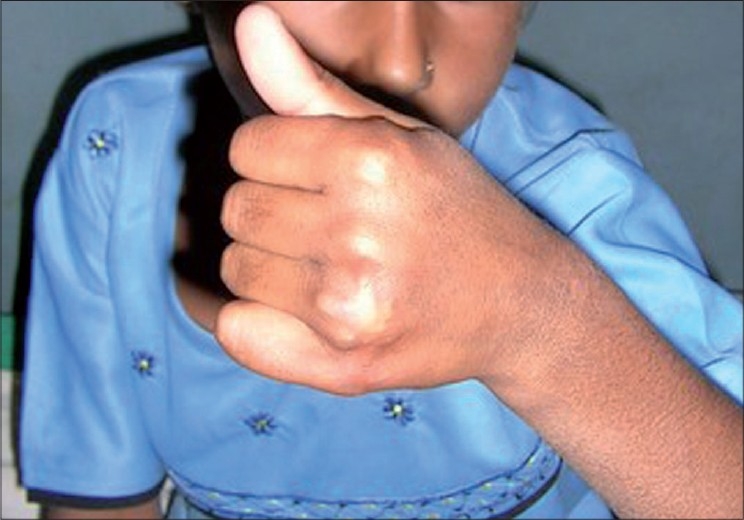
Six-year-old male with fodder-cutting machine injury with cut extensors to ring and little finger (Zone V). He also had fracture of head of V metacarpal

At the end of six weeks, five patients showed a mild extensor lag (good result). After extensor strengthening exercises the patients were again assessed and results were now excellent, i.e. no extensor lag. In two patients the results were poor. Out of these two patients, one patient had metacarpal loss at the time of injury and the other patient had a metacarpal fracture [Figure 5] and associated flexor tendon injury in the same hand. He was unable to carry out the exercise protocol adequately.

In the series of Sylaidis, Youatt and Logan,^13^ 24 patients were followed for up to six weeks, 92% (*n* = 22) showed excellent/good results. The splint used and the rehabilitation protocol used by them was similar to ours. Our results are comparable with this series. Newport *et al.*,^8^ examined the long-term results of extensor tendon repair in 101 digits treated with traditional static splinting. The complex injuries achieved 45% good or excellent results. Both, simple injuries and those with the addition of joint capsule injury only achieved 64% good to excellent results. Our results are also comparable with rehabilitation using dynamic splinting by Cosby and Wehbe^3^ who had 92% good to excellent results in injuries on Zones IV to VII.

The early mobilization of repaired extensor tendons, prevents formation of adhesions as compared to rigid immobilization. The static splint besides being easy to prepare and apply gives equally good results as the dynamic splints. The patient compliance with this easy-to-follow rehabilitation plan was very good, thus helping to attain excellent results. The patients return to work early, thus reducing the amount of workdays lost.

## References

[1] Tuncali D, Yavuz N, Terzioglu A, Aslan G (2005). The rate of upper-extremity deep-structure injuries through small penetrating lacerations. Ann Plast Surg.

[2] Hague MF (1954). The results of tendon suture of the hand: A review of 500 patients. Acta Orthop Scand.

[3] Crosby CA, Wehbé MA (1999). Early protected motion after extensor tendon repair. J Hand Surg.

[4] Chow JA, Dovelle S, Thomes LJ, Ho PK, Saldana I (1989). A comparison of results of extensor tendon repair followed by early controlled mobilization versus static immobilization. J Hand Surg Br.

[5] Slater RR, Bynum DK (1997). Simplified functional splinting after extensor tenorrhaphy. J Hand Surg Am.

[6] Evans RB (1986). Therapeutic management of extensor tendon injuries. Hand Clin.

[7] Evans RB, Burkhalter WE (1986). A study of the dynamic anatomy of the extensor tendons and implications for treatment. J Hand Surg Am.

[8] Newport ML, Shukla A (1992). Electrophysiological basis of dynamic ext. Splinting J Hand Surg Am.

[9] O'Dwyer FG (1990). Quinton DN. Early mobilization of acute middle slip injuries. J Hand Surg Br.

[10] Rolph-Roeming K (1992). Early mobilization of extensor tendon lacerations in ZoneIII and IV. J Hand Ther.

[11] Saldana MJ, Chobban S (1991). Results of acute Zone III extensor tendon injuries, Treatment by dynamic splinting. J Hand Surg Am.

[12] Kleinert HE, Verdan C (1983). Report of the committee on tendon injuries. J Hand Surg.

[13] Sylaidis P, Youatt M, Logan A (1999). Early active mobilization for extensor tendon injuries. J Hand Surg Br.

[14] Dargan EL (1969). Management of extensor tendon injuries of the hand. Surg Gynecol Obstet.

[15] Kelly AP (1956). Primary tendon repair: A study of 789 consecutive tendon severances. J Bone Joint Surg Am.

[16] Zander CL (1987). The use of early mobilization following complex injury to the extensor tendons. J Hand Ther.

[17] Pandey VK, Goyal A (1988). Study of Extensor Injuries of hand and wrist. Indian J Orthop.

[18] Flynn JE, Graham JH (1962). Healing following tendon suture and tendon transplants. Surg Gynaecol Obstet.

[19] Mason ML, Shearon CG (1932). The process of tenon repair. Arch Surg.

[20] Gelberman RH, Amifl D, Gonsalves M, Woo S, Akeson WH (1981). The influence of protected passive mobilization on the healing of flexor tendons: A biochemical and microangiographic study. Hand.

[21] Bunnell S (1951). The early treatment of hand injuries. J Bone Joint Surg Am.

[22] Blue AT, Spira M, Hardy SB (1976). Repair of Extensor tendon injuries of the hand. Am J Surg.

[23] Lister GD, Kleinert HE, Kutz JE, Atasoy E (1977). Primary flexor tendon repair followed by immediate controlled mobilization. J Hand Surg.

